# Consumption of Ready-to-Eat Cereal in Canada and Its Contribution to Nutrient Intake and Nutrient Density among Canadians

**DOI:** 10.3390/nu11051009

**Published:** 2019-05-03

**Authors:** Hassan Vatanparast, Naorin Islam, Rashmi Prakash Patil, Arash Shamloo, Pardis Keshavarz, Jessica Smith, Luan Manh Chu, Susan Whiting

**Affiliations:** 1 School of Public Health, College of Pharmacy and Nutrition, University of Saskatchewan, Saskatoon, SK S7N 4Z2, Canada; naorinislam7@gmail.com (N.I.); rashmi.patil@usask.ca (R.P.P.); arash.shamloo@usask.ca (A.S.); pak526@mail.usask.ca (P.K.); susan.whiting@usask.ca (S.W.); 2 Bell Institute of Health and Nutrition, General Mills, Minneapolis, MN 55427-3870, USA; Jessica.Smith@genmills.com; 3 Canadian Centre for Health and Safety in Agriculture (CCHSA), University of Saskatchewan, Saskatoon, SK S7N 4Z2, Canada; cml779@mail.usask.ca

**Keywords:** ready-to-eat cereal, nutrient intake, dietary assessment, nutrient density

## Abstract

In recent years, ready-to-eat cereal (RTEC) has become a common breakfast option in Canada and worldwide. This study used the nationally representative cross-sectional data from the Canadian Community Health Survey (CCHS) 2015-Nutrition to determine patterns of RTEC consumption in Canada and the contribution to nutrient intake among Canadians who were ≥2 years, of whom 22 ± 0.6% consumed RTEC on any given day. The prevalence of RTEC consumption was highest in children aged two to 12 years (37.6 ± 1.2%), followed by adolescents aged 13 to 18 years (28.8 ± 1.4%), and then by adults ≥19 years (18.9 ± 0.6%). RTEC consumers had higher intakes of “nutrients to encourage” compared to the RTEC non-consumers. More than 15% of the daily intake of some nutrients, such as folic acid, iron, thiamin, and vitamin B_6_, were contributed by RTEC. It was noted that nearly 66% of milk consumption was co-consumed with RTEC among RTEC consumers. The nutrient density of the diet, as defined by Nutrient-Rich Food Index (NRF 9.3), was significantly higher among RTEC consumers compared to non-consumers. RTEC consumption was not associated with overweight/obesity. RTEC consumption considerably contributed to the intake of some key nutrients among all age groups in Canada.

## 1. Background

In recent years, Canada has seen significant changes in dietary patterns, with more Canadians consuming diets that are excessive in energy intake but inadequate in nutrients and key food groups including fruit, vegetables, whole grains, dairy, and fiber [1,2]. Poor dietary habits may contribute to an increased risk of overweight or obesity and other chronic diseases among children and adults [3]. Overweight/obesity is of increasing public health concern in Canada with the rates of 23% and 54% in adolescents and adults, respectively [4]. These differences in rates may be attributed to some factors, including socioeconomic status and lifestyle behaviors, such as dietary intakes, eating habits, and physical activity.

Ready-to-eat cereal (RTEC) is a popular breakfast option in many countries, including Canada, and the majority of RTEC consumption occurs at breakfast. RTECs are defined as any processed cereal that can be eaten without further preparation [5]. However, RTEC products can be high in added sugar due to commercial purposes, and a RTEC breakfast supplies more glucose and fructose in comparison to a bread breakfast, but overall it would not lead to a higher consumption of simple sugars over a full day [5]. RTECs are typically grain-based (often whole grain), with formulations consisting mainly of rice, corn, wheat, and oats. There are over 160 varieties of ready-to-eat cereals available across Canada, many of which are fortified with vitamins and minerals, including folic acid, thiamin, niacin, pantothenic acid, vitamin B_6_, zinc, and iron [1,6]. Generally, RTECs are associated with better overall diet such as lower fat and a higher fiber intake, and improved body mass index outcomes [7].

Shifts are occurring in nutrition research and dietary recommendations to focus more on food-based, rather than nutrient-based, recommendations. Therefore, further research is needed to understand the associations between specific foods and dietary and health outcomes. Measures of overall diet quality can also be particularly useful. The Nutrient-Rich Food Index 9.3 (NRF 9.3) applies to individual foods, but can also be used to measure the nutrient density of the overall diet, thus making it a useful tool in assessing the nutrient density of individuals’ diets based on their intakes [8].

In order to determine the role of RTEC in the Canadian diet, the recent nationally representative dietary survey, the Canadian Community Health Survey (CCHS) 2015, had been used to evaluate how RTEC consumption contributed to daily energy and nutrient intakes, and then compare them with non-consumers, as well evaluating the contribution of milk co-consumed with RTEC to total daily milk consumption. The other objective is to represent the association of RTEC with diet quality (measured using the NRF 9.3 applied to the total diet) nationally and provincially, and health status of Canadians by age, sex, region of residence, and socioeconomic status.

## 2. Subject and Method

### 2.1. Data Source and Analyical Sample

This descriptive, cross-sectional study was carried out by analyzing data obtained from CCHS 2015 Nutrition; a cross-sectional, nationally, and provincially representative survey of Canadians at national and provincial levels. The target population for CCHS 2015-Nutrition included all individuals aged one year and older, living in private dwellings in the 10 Canadian provinces. The survey conducted dietary assessment via two 24-h recall, alongside a general health questionnaire accounting for sociodemographic characters such as food security, age, sex, immigration status, smoking, physical activity, and measured height and weight body mass index (BMI). The final sample size was 20,487 for day 1 of the 24-h recall. The overall response rate was 62% at the national level, representing about 98% of the Canadian population. Detailed information about the methodology of data collection in CCHS 2015 can be found elsewhere [9,10]. The detailed data files are available at the Statistics Canada Research Data Center (RDC) across the country. To access the data, a proposal should be submitted after approval and the permission to access data is provided, and the analysis can be done only inside the Statistics Canada Research Data Center (RDC) [11]. The results were submitted to the RDC data analyst for vetting purposes. However, to comply with Statistics Canada data vetting regulations, data had been aggregated in larger groups in some cases.

#### 2.1.1. Dietary Data Collection

The automated multiple-pass method (AMPM) was used to conduct 24-h recall. Data were collected on the amount, type, time, occasion, and location of food consumed in the past 24 h [12]. The first recall, i.e., day 1 of the 24-h recall, was conducted using a computer-assisted personal interview, and the second recall i.e. day 2 of the 24-h recall, was conducted using a computer-assisted telephonic interview. Proxy interviews were carried out for children aged one to six years. For children aged six to 11 years, data was collected via parents’ assistance, and for individuals 12 years and above, a non-proxy method of collection was applied. Approval from the Canadian Research Data Center Network and Statistics Canada was obtained to grant access to data for the analysis documented in this paper [13]. In this study, only the data collected from day 1 of the 24-h recall had been used.

The population representation for the current research includes 19,677 Canadians aged two years and older, with valid 24-h recall on day 1. A valid recall was defined as an energy intake within 200–8000 kcal/day, as we used in our other studies [2]. Individuals who were pregnant or lactating were excluded from the study sample. The extreme outliers with unrealistically high intakes of food groups and nutrients were also excluded. Low intakes were not excluded as outliers, since it is possible not to take a particular nutrient or food group in a particular day. Participants were divided according to their age—children 2–12 years (*n* = 3810), teens 13–18 years (*n* = 2379), and adults ≥19 years (*n* = 13,577)—and RTEC consumption status. An individual was defined as an RTEC consumer if they reported consuming any amount, at any meal occasion, of RTEC on day 1 of the 24-h recall. In this study, 5027 individuals reported ready-to-eat cereal consumption. In instances where the sample size would be too small, the children and teen age group had been pooled, and reported results according to two age groups: all children 2–18 years and adults ≥19 years.

#### 2.1.2. Descriptive Characteristics

Several descriptive characteristics by age and RTEC consumption status including sex, smoking status (yes, no), ethnicity (% Caucasian), education (university degree or lower), marital status (yes, no; for adults), food security (secure, insecure), immigrant (yes, no), BMI (adults), BMI z-score (children), prevalence of overweight/obesity, and prevalence of residence in an urban setting had been reported. The BMI z-score for children age 5 to 18 years was calculated following the protocol from the World Health Organization (WHO) using the macro that is available on their website [14]. Their provided SAS macro estimates the BMI z-score for children 5–19 years [14]. For adults, overweight/obesity categories and BMI as a continuous variable were available in the CCHS data [15]. The Canadian provinces had been cateforized into five regions, including Atlantic (Newfoundland, Labrador, Nova Scotia, and New Brunswick), Ontario, Quebec, Prairies (Manitoba, Saskatchewan, and Alberta), and British Columbia, to avoid the small cell sizes. The location of each eating occasion of RTEC was reported. To avoid a small proportion of participants, the location of RTEC consumption had been categorized into two levels: “home” and “away from home”. The categorical sociodemographic variables included sex, smoking (yes, no), ethnicity (% Caucasian), education (university degree or lower), marital status (yes, no), food security (secure, insecure), overweight/obese (yes, no), residence (urban, rural), and immigrant (yes, no), and age groups. The sociodemographic differences between RTEC consumers and non-consumers were reported by two age groups (2–18 years and ≥19 years).

#### 2.1.3. Daily Nutrients and Energy Intake

The intakes of nutrients and energy from food between RTEC consumers and non-consumers had been compared. Further, the contribution of RTEC to the daily intake of nutrients was computed. Results were adjusted for ethnicity, smoking, education, food security, age, immigrant, and daily energy intake (kcal/day).

#### 2.1.4. Consumption of Milk and RTEC

To determine the co-consumption of milk with RTEC, the intake of both food items had been cross-matched with the reported time of consumption. The percent contributions of milk consumed with RTEC to daily milk consumption were obtained for the entire population and the three age groups (children 2–12 years, teens 13–18 years, and adults 19 years and older). At the regional level, the data had been presented by two age groups (2–18 years and ≥19 years).

#### 2.1.5. Diet Quality Index

The NRF 9.3 index has been used to measure the nutrient density of the daily dietary intake of RTEC consumers and non-consumers [16] as an indicator of overall diet quality. The NRF 9.3, when used to assess overall diet, has previously been shown to correlate with other established measures of diet quality (such as the Healthy Eating Index) and to be associated with positive health outcomes [16]. An advantage of the NRF 9.3 is that it does not require quantitative disaggregated food group data to calculate, which was not available from CCHS 2015 at the time of this analysis. The NRF 9.3 index used in this study is a slightly modified version of the original method. It is calculated by the sum of the percentage of daily values (DVs) of nine nutrients to encourage per 2000 kcal including protein, fiber, vitamin A, vitamin C, vitamin E, calcium, iron, magnesium, and potassium, minus the sum of the percentage of the maximum recommended values for three nutrients, which were limited to including saturated fat, added sugar, and sodium [2]. Since the CCHS 2015 data does not contain data on Vitamin E intake and added sugar, the former was replaced with Vitamin D, and the latter was replaced by total sugars in the current study. For each of the nine nutrients that were encouraged, percentage DVs were abridged at 100 for intakes equal or above the DV. For each of the three nutrients to limit the percentage, the DV was expressed as the percentage by which intake exceeded the DV (and accordingly, was zero when intake was ≤DV). The maximum possible score was 900 points, reflecting a diet in which intakes per 2000 kcal were ≥DV for all nine nutrients that were encouraged and ≤DV for all three nutrients to limit. This study used the recently updated Canadian DVs for nutrient intakes [17]. The higher the score, the more nutrient-dense the diet.

### 2.2. Method

All statistical analyses were carried out using SAS (Version 9.4, SAS Institute, Toronto, Canada) software at the Sky Research Data Center—University of Saskatchewan. The weighting and bootstrapping procedure recommended by Statistics Canada was applied to the entire analysis to procure population-level estimates. Values are represented in means (SE) and percentages (SE) where applicable. The chi-squared test had been used to compare the distribution of categorical sociodemographic variables between RTEC consumers and non-consumers. The Student t-test was used for comparing continuous variables between RTEC consumers and non-consumers. Variables that differed significantly between RTEC consumers and non-consumers were included as covariates (ethnicity, education, smoking, food security, age, immigration, and energy intake) in regression models comparing daily energy and nutrient intakes. An analysis of covariance (ANCOVA) statistical test was used to determine the differences across age groups in mean daily energy and nutrient intakes at breakfast and daily, and the proportions of nutrients contributed from RTEC to daily intakes. A similar test was used to obtain the differences of daily energy and nutrient intake and proportions of nutrient contribution between RTEC consumers and non-consumers. For post-hoc tests, a Hsu-adjusted α = 0.025 was applied [18]. Patterns of milk consumption with RTEC were measured using a simple F-test. Alpha was set at 0.05.

## 3. Results

### 3.1. Prevalence of RTEC Consumption at the National Level

On a given day, 22 ± 0.6% of Canadians reported consuming RTEC. The consumption of RTEC among women was 22.2 ± 0.75%, and among men was 21.6 ± 0.78%. The prevalence of RTEC consumption was significantly higher in children aged 2–12 years (37.6 ± 1.22%) followed by adolescents aged 13–18 years (28.8 ± 1.42%), and then by adults ≥19 years (18.9 ± 0.64%). Overall, 92.5 ± 0.81% of Canadians reported consuming RTEC at home. Mean grams of RTEC consumption was 30.2 ± 0.88 g/day for children aged 2–12 years, 47.3 ± 2.32 g/day for adolescents aged 13–18 years, and 43.9 ± 1.07 g/day for adults ≥19 years. Moreover, RTEC mean intake (gram) in a day was the highest among adolescents (13–18 years) compared to children (2–12 years) and adults (≥19 years).

Table 1 provides information about the sociodemographic characteristics of cereal consumers and non-consumers. Among children, RTEC consumers were significantly younger, less likely to smoke, and more likely to be of Caucasian ethnicity. Among adults, RTEC consumers were significantly older, less likely to smoke, more likely to be Caucasian, less likely to be a university graduate, and less likely to be an immigrant to Canada.

### 3.2. Prevalence of RTEC Consumption at Region Level

The percent of RTEC consumers across sex and age groups are presented for the five Canadian regions in Table 2. The Atlantic region had the highest rate of RTEC consumers, with 24.4 ± 1.0% when compared to other regions, which ranged between 21.2 ± 1.2% in Ontario and 22.1 ± 1.0% in the Prairies, with no differences among these four regions. Among children aged 2–18 years, the percent consumers was the highest in Prairies at 36.1 ± 1.8%, with the lowest being the Atlantic region at 25.2 ± 1.4%. Among adults aged ≥19 years, the percent consumers was the highest in Quebec with 19.0 ± 1.4% and lowest for the Atlantic region at 15.2 ± 0.4%.

### 3.3. Daily Energy and Nutrient Intake Comparison between RTEC Consumers and Non-Consumers

Table 3 displays the mean energy and nutrient intakes of RTEC consumers and non-consumers for the Canadian population and for the three age groups. Overall, for the total population, there was no difference in the total daily energy intake between RTEC consumers and non-consumers. Intakes of fiber, carbohydrate (g), percentage of energy from carbohydrates, and total sugar (g) were significantly higher in RTEC consumers compared to non-consumers. In all the age groups, the intakes of vitamins and minerals, including vitamin B_12_, vitamin B_6_, folate, vitamin D, riboflavin, thiamin, potassium, zinc, calcium, iron, and magnesium were significantly higher in RTEC consumers in comparison to non-consumers. Sodium consumption was significantly lower among RTEC consumers compared to non-consumers. This pattern of differences was consistent across age groups, although there were some notable exceptions. For example, there was no difference in total sugar intake; saturated fat intake was lower among children who consumed RTEC compared to those who did not consume RTEC.

### 3.4. Nutrient Contribution of Ready-to-Eat Cereal to Daily Nutrient Intake

**Figure 1:**
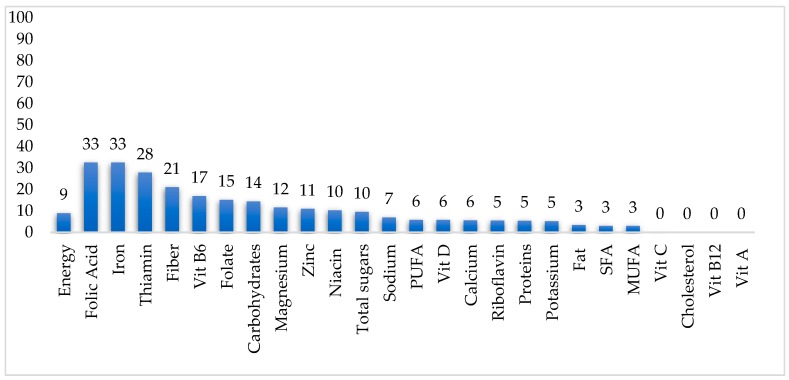
Contribution (%) of ready-to-eat cereal (RTEC) to daily nutrient intake among RTEC consumers (*n* = 7,354,198) ^1^. Data source: Canadian Community Health Survey, Nutrition-2015. ^1^ All data are weighted and bootstrapped to obtain estimates at the Canadian population level. RTEC consumers were defined as those individuals who reported any quantity of ready-to-eat cereal consumption on day 1 of the 24-h recall. MUFA: monounsaturated fatty acids, PUFA: polyunsaturated fatty acids, SFA: saturated fatty acids, Vit: vitamin.

The percentage nutrient contribution of RTEC to daily nutrient intake for Canadians aged 2 years and over who consume RTEC are shown in Figure 1. Table 4 represents similar data broken down into three age groups: 2–12 years, 13-18 years, and ≥19 years. In Figure 1, among Canadians aged 2 years and over, RTEC contributed 32.5% of daily folic acid and iron, 27.9% of thiamin and 21% of fiber intake. RTEC also contributed 10% to 17% daily intake of vitamin B_6_, dietary folate equivalent, carbohydrates, magnesium, zinc, and niacin. The figure also represents the 9.5% daily total sugar intake contributed by RTEC. Moreover, RTEC contributed only 8.9% of daily energy intake. Total sugar intake was approximately proportional (±1%) to energy intake, contributing 9.5% to total intake. When examined across the three age groups (Table 4), the energy contributions from RTEC to daily energy intake was significantly different across the age groups. Energy contributions for children aged 2–12 years at daily energy was 7.59%; for adolescents aged 13–18 years, RTEC contributed to 9.36% of daily energy intake (the highest), and for adults aged ≥19 years, RTEC contributed to 9.26% of daily energy intake. However, the pattern of the contribution of nutrients to daily intakes was similar across age groups. The only exception was that total sugar intake for teens was proportionally higher (10.5%) than the total energy intake (9.4%), and zinc intake was similar to energy intake (10.3%).

### 3.5. Contribution of Ready-To-Eat Cereal Co-Consumed with Milk to Nutrients at Breakfast and Daily

**Figure 2:**
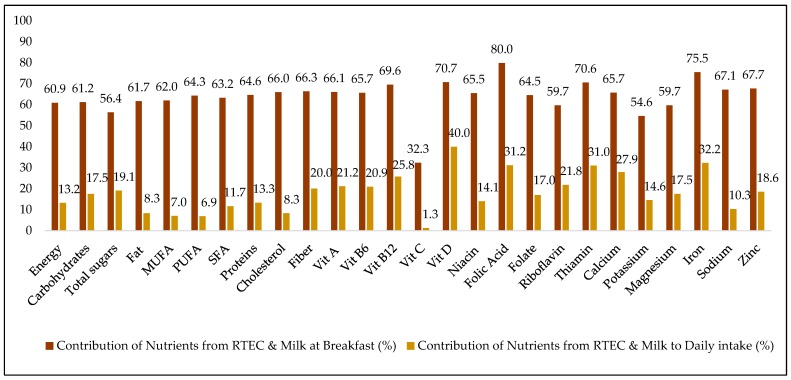
Contribution (%) of ready to eat cereal (RTEC) and milk to nutrient intake at breakfast and daily among all Canadians ^1^. ^1^ All data are weighted and bootstrapped to obtain estimates at the Canadian population level. Data source: 2015 Canadian Community Health Survey-Nutrition. Co-consumption was determined when the period reported for consumption of ready-to-eat cereal and milk was the same. MUFA: monounsaturated fatty acids, PUFA: polyunsaturated fatty acids, SFA: saturated fatty acids, Vit: vitamin.

Figure 2 presents the contribution of nutrients from RTEC and milk when co-consumed at breakfast to daily nutrients. Overall, RTEC and milk contributed to 60% of energy at breakfast and 13% of total daily energy among the people who co-consumed RTEC and milk. Compared to contribution to energy intake at breakfast and daily, the co-consumption of RTEC and milk had higher contribution to the intake of folic acid, folate, iron, vitamin D, thiamin, vitamin B_12_, zinc, sodium, folate, calcium, vitamin B_6_, vitamin A, fiber, cholesterol, proteins, SFA, PUFA, MUFA, fat, and carbohydrates.

### 3.6. Contribution of Milk Consumed along with RTEC to Daily Milk Consumption

**Figure 3:**
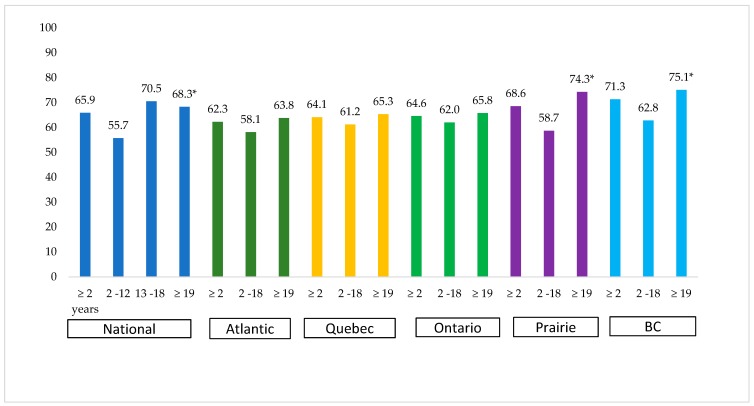
Contribution (%) of milk consumed along with ready-to-eat cereal to daily milk intake among the people who consumed RTEC and milk at the same time (*n* = 5,839,304) across age groups and at national and regional levels ^1^. Data source: 2015 Canadian Community Health Survey-Nutrition. ^1^ All data are weighted and bootstrapped to obtain estimates at the Canadian population level. Co-consumption was determined when the period reported for ready-to-eat cereal consumption and milk was the same. *Percentage differences of milk contribution, consumed with ready-to-eat cereal across age groups 2–12 years, 13–18 years, and ≥19 years at a 0.05 level of significance. Weighted frequency: national level: ≥2 years: 5,839,304, 2–12 years: 1,211,317, 13–18 years: 557,941, ≥19 years: 4,070,046; regional level: Atlantic—2–18 years: 113709, ≥19 years: 318,400, Quebec—2–18 years: 398,058, ≥19 years: 968,932, Ontario—2–18 years: 695,603, ≥19 years: 1,511,113, Prairie—2–18 years: 402,290, ≥19 years: 697,270, British Columbia—2–18 years: 225,830, ≥19 years: 508,099. BC: British Columbia.

At the national level, the contribution of milk consumed with RTEC to total daily milk consumption was 65.9% (Figure 3). Among the subgroups, 13 to 18-year-old adolescents had the largest contribution (70.5%), followed by adults ≥19 years with 68.3%, and 55.7% for children 2–12 years. Regional data showed that in adults ≥19 years, almost all the regions had the contributions of milk co-consumed with RTEC to make up more than 63% of daily milk consumption. Significant differences in milk consumption with RTEC was noted within age groups in British Columbia and the Prairies.

### 3.7. Differences in Nutrient Density among RTEC Consumers and Non-Consumers

Table 5 presents the NRF 9.3 scores of RTEC non-consumers and consumers across all ages and within three age groups, as well as the regional level among all Canadians and across two age groups. The NRF 9.3 scores were significantly higher across all age groups in RTEC consumers compared to non-consumers provincially and nationally. At the national level, adolescents between 13–18 years had the highest NRF 9.3 scores with a significant difference in score compared to non-consumers (569.7 ± 4 versus 505.6 ± 2.4). Among the ≥2 age group, the NRF 9.3 score was 567.5 ± 4.5, which was higher than that of the non-consumers, which was 514.0 ± 3.8. At the regional level, British Columbia had the highest NRF 9.3 score among ≥19 years adults, 584.4 ± 11.3; it was followed by Ontario (578.7 ± 7.3), Atlantic (567.5 ± 6.6), Quebec (562.8 ± 9.2) and Prairies (553.6 ± 8.8). A similar pattern was seen for the other age groups across regions, except for Quebec and Atlantic (the NRF 9.3 score of Quebec was greater than that of the Atlantic).

### 3.8. Comparison of BMI between RTEC Consumers and Non-Consumers

No significant difference was found between the BMI of RTEC consumers and non-consumers among both children and adults. For adults, RTEC consumers had a mean BMI of 27.4 ± 0.2, and non-consumers had a mean BMI of 27.4 ± 0.1. For children, RTEC consumers had a mean BMI z-score of 0.51 ± 0.06, and non-consumers had a BMI z-score of 0.44 ± 0.05.

### 3.9. Comparison of Grain Consumption between RTEC Consumers and Non-Consumers

Table 6 represents the percent of total grain consumption with RTEC among all the Canadian RTEC consumers, which was broken over three age groups. RTEC contributed to 31% of total grain consumption. In Canada, 63% of whole grain consumption was from RTEC. Adults (≥19 years) had the highest consumption of grains from RTEC with significant difference between other two age groups.

## 4. Discussion

To our knowledge, this is the first study showing that Canadian RTEC consumers had more nutrient-dense diets. RTEC is a popular food in Canada, with approximately 22% of Canadians consuming RTEC, particularly among children, who are the highest consumers of RTEC compared to adolescents and adults. It had been found that RTEC consumers, compared to non-consumers, had higher daily intakes of key micronutrients including vitamin B_12_, B_6_, folate, vitamin A, vitamin C, vitamin D, calcium, magnesium, zinc, and phosphorous, which are considered as shortfall nutrients [19]. This may be due in part to the healthier overall diets of RTEC consumers, but RTEC itself—either alone or combined with milk—contributed key nutrients to the diets. Additionally, frequent RTEC consumption was associated with better nutrient intake profiles and higher whole grain intake from RTEC among RTEC consumers in different age groups. Lastly, we found that there was no significant differences between BMI among RTEC consumers and non-consumers.

The rate of RTEC consumption varies across the globe. Our findings indicated that over one-fourth of Canadians were RTEC consumers, and the prevalence of RTEC consumption was higher among 2 to 12-year-old children (37.6%) compared to the other age groups. Over 26 years ago, Sommerville and O’Reagan reported that 86.4% of Ireland’s population between 8–18 years were RTEC consumers [20]. In 1993, Crawley found that in the United Kingdom, 78.7% of males and 63.1% of females aged 16–17 years old consumed RTEC [20]. In Greece, 26.9% of the adolescents consumed RTECs regularly, and 43% of them consumed at least once a week [21]. However, a relatively recent study reports around 65% of European adolescents who participated in a study in nine countries consumed RTEC at least once a week [5]. Despite some variability across region and time, RTEC has been and continues to be a popular dietary choice, particularly among children.

Studies have suggested that the consumption of RTEC contributes to a balanced diet with a lesser proportion of energy provided by fats and a higher proportion of fiber and carbohydrate intake [22]. However, one cannot ignore the added sugar content that might make RTEC more pleasant and tasty to eat [22]. In our study, the daily fat intake was lower, while the intakes of vitamin B_12_, vitamin B_6_, folate, riboflavin, thiamin, sodium, potassium, zinc, magnesium, iron, and calcium were higher among RTEC consumers compared to non-consumers. RTEC consumers’ total sugar intake was higher than non-consumers in the total population, and for the other age groups as well. Total sugar intake did not differ between children who ate RTEC and those who did not. However, the CCHS dataset does not contain information on added sugar intake, so it is possible that dietary sources other than RTEC are contributing to this total sugar intake, including higher fruit intake and higher milk intake [2]. When we looked specifically at the contribution that RTEC consumed with milk made to the diet, it had been found that RTEC contributed 20% to 40% of the daily intake of fiber, vitamin B_6_, vitamin A, vitamin B_12_, calcium, thiamin, folic acid, iron, and vitamin D. RTEC and milk combined contributed to 19% of the total sugar intake but alone (without milk), RTEC contributed to 9.5% of the total sugar intake, which is proportional to its contribution to energy intake (8.9%).

A large proportion, 66%, of milk consumed in a day was consumed with RTEC, particularly for adolescents (71% of milk). Milk consumption with RTEC was highest among adults ≥19 years across all the regions, but was highest in British Columbia, where approximately 3/4 of all milk consumption is with RTEC.

In Canada, it is permitted to fortify cereal breakfast with the type and amount of micronutrients that are specified by Health Canada, including thiamine, niacin, vitamin B_6_, folic acid, pantothenic acid, magnesium, iron, and zinc [1]. Hence, it is not surprising, to find these significant differences. The higher vitamin D intake in RTEC consumers is probably because of co-consumption with milk through mandatory vitamin D fortification in Canada, and overall healthier dietary choices. It is not common to fortify RTEC items with vitamin D. Similar results have been previously reported in the United States (USA) [23], where studies found that RTEC consumer adults and children had a lower daily intake of total fat, saturated fatty acids, and cholesterol [24]. In the Bogalusa Heart Study [25], adults consuming RTEC also had higher intakes of total carbohydrates, both starch and total sugars, than those who did not eat cereals (who consumed a higher intake of fat) as we found in our study. In another population-based survey of Canadians aged 12 years and older in 2004, the mean intakes of thiamin, riboflavin, niacin, folic acid, vitamin A, vitamin B_6_, vitamin B_12_, and iron were higher among RTEC consumers [1]. The same phenomenon has been observed in Albertson’s 2013 study [1] in Canada, which demonstrated that those who consumed RTE cereal (participants age 55 and older in 2008) most often had improved micronutrient intakes and were more likely to meet the dietary reference intake standards, compared with those consumed RTEC less often or not at all. These results are in agreement with the past research conducted in the USA [6,25,26,27,28,29,30,31], reflecting the significant contribution of RTEC to nutrient intake and diet quality. The improved nutrient intake profile seems to be largely related to the consumption of RTEC, the foods that RTEC could be replacing, and a pattern for healthful eating throughout the day.

On the other hand, our findings in terms of lower sodium intake in RTEC consumers was different from the findings by Susan Barr in 2013 [30], who reported no significant difference in sodium intake between RTEC consumers and non-consumers. This is likely due to the decreasing trend toward sodium levels in RTEC (in the case of Canada). This decline might be owing to Health Canada’s recent efforts that showed that voluntary sodium reduction in processed foods accounted for a decrease of 8% in average daily sodium intake between 2010–2016. According to the report on Sodium Reduction in Processed Foods report [32], the sodium reduction in Canada for RTEC has been conducted in three phases from 2012 to 2017. Initially, the baseline level was 558 mg sodium per 100 g of RTEC, and it reduced to 490 mg/100 g, 430 mg/100 g, and 360 mg/100 g in phases I, II, and III, respectively. Although the measured level should be 395 mg/100 g of RTEC in 2017, it is reported that Phase-II targets were met, which means that RTEC have partially met the sodium reduction goal [32].

The total grain consumption from RTEC among RTEC consumers was higher among ≥19-year-old adults who consumed nearly 33% of grain via RTEC, of which approximately 64% was allocated to the whole grain among RTEC consumers. To our knowledge, there is no study reporting the proportion of whole grain consumption from RTEC among consumers in Canada. The previous version of Canada’s Food Guide recommends three to eight servings/day (age and sex-dependent) of grain products, and advises making at least one-half of the grain product choices whole grain each day [19]. The minimum content of whole-grain consumption is recommended to be 8 g of whole grain per 30 g of cereal (in other words, 27 g of whole grain per 100 g) [33]. Canada’s Food Guide recommends 30 g in cold cereal and 150 g in hot cereal [34]. Canada’s new dietary guidelines are encouraging whole grain intake as the primary source of the grain products [34]. Considering the proportion of Canadians consuming RTEC, particularly children and adolescents, RTEC can be considered one of the means for promoting whole grain consumption.

The NRF 9.3 index is preferred for evaluating the nutrient density and diet quality, because it includes the need to encourage nutrients of public health importance such as proteins, fibers, vitamin A, vitamin C, vitamin D, calcium, iron, magnesium, and potassium, and to limit fat, added sugars, and sodium [35]. The NRF 9.3 index can be applied to individual foods, meals, and total diets, and diets assigned higher NRF scores were associated with a higher consumption of foods and nutrients to encourage and lower energy-dense foods [35]. The observed results for NRF scores between RTEC consumers and RTEC non-consumers indicated that RTEC consumption increased the nutrient-rich food score across all age groups. Similar to data from the USA [31], adults aged ≥19 years old had the highest score intake of RTEC among all the age groups. At the provincial level, adults in British Columbia had the highest score. The higher contribution of milk consumption along with RTEC to this province may explain the highest score. No similar study was available in other countries to compare with the results of our study.

In addition to contributions to research and informing the public, this study may provide the food industry with a general picture of RTEC nutrients and daily energy intake contribution in order to alter their products to be more healthy and beneficial. This study also would benefit the monitoring bodies to track the RTEC consumption nationally and regionally based on their policies.

Our study used CCHS 2015 Nutrition data, which is a comprehensive nutrition survey and represents all Canadians. Over-reporting was identified during analysis, and nutrients with an unrealistic higher intake were excluded. The other strengths of our study include using measured BMI (not self-reported) for anthropometry measurements, adjusting for key confoundings, and reporting the results at the national and regional level. The limitations are that the cross-sectional design of the study does not allow any causality inference. Our study included information based on one day of self-reported 24-h recall, which is subject to over and under-reporting, and could possibly not represent usual food or nutrient intake along with weekend food consumption. Although the mean of one-day intake in national survey data may be similar to data from two days of 24-h recall (collected in national surveys), using data from serial 24-h recall would provide more robust estimates of usual intake. A modified NRF 9.3 index had been used with some modifications as described in our methods, because data on vitamin E and added sugar were not available in the dataset. For recognizing the diet quality, in this study, total sugar was considered instead of added sugar, which prevented us from identifying the exact amount of sugar consumption that was added to food during their production. Hence, providing data on added sugar in national surveys would be beneficial.

## 5. Conclusions

RTEC, as a popular breakfast item, is consumed by over one-fifth of Canadians. Relative to its contribution to daily energy (8.9%), RTEC provided considerable amounts of nutrients such as iron, thiamin, vitamin B_6_, and folate. Milk and RTEC when co-consumed contributed to relatively higher amounts of other key nutrients such as calcium and vitamin D. In the light of the new Canada’s dietary guidelines, the contribution to whole grain intake, the higher overall diet quality, and lack of association with overweight and obesity, suggests whole grain RTEC as a healthy food choice for Canadians.

## Figures and Tables

**Table 1:** Sociodemographic Characteristics of Canadian Children and Adults among Ready-to-Eat Cereal (RTEC) Consumers and Non-Consumers ^1^.

Characteristics	Children and Teens (2–18 years) (*n* = 6,463,895)		Adults ( ≥19 years) (*n* = 27,169,337)	
	RTEC Consumers	RTEC Non-Consumers	RTEC Consumers	RTEC Non-Consumers
Mean age ± SE (year)	9.1 ± 0.1	10.3 ± 0.1 *	52.7 ± 0.6	48.5 ± 0.2 *
Sex (% male)	50.7 ± 1.6	50.0 ± 1.1	48.7 ± 1.6	50.2 ± 0.4
Smoker (% yes) ^2^	2.1 ± 0.8	4.6 ± 0.6 *	15.3 ± 1.5	19.5 ± 0.8 *
Ethnicity (% Caucasian)	70.2 ± 1.9	66.1 ± 1.5 *	85.3 ± 1.3	72.6 ± 1.1 *
Education (% university grad) ^3^	44.5 ± 1.8	44.5 ± 1.4	34.7 ± 1.9	39.5 ± 1.0 *
Marital status (% married or co-habiting) ^4^	n/a	n/a	63.9 ±1.7	64.1 ± 1.0
Food secure (% yes)	85.0 ± 1.3	83.5 ± 1.0	90.8 ± 1.0	88.1 ± 0.6
BMI (kg/m^2^)	n/a	n/a	27.359 ± 0.2	27.358 ± 0.1
BMI z-score (≥5 years and older)	0.51 ± 0.06	0.44 ± 0.05	n/a	n/a
Overweight/obese (% yes) ^5^	27.0 ± 1.9	26.2 ± 1.2	62.9 ± 1.8	61.6 ± 1.2
Urban residence (% yes)	82.8 ± 1.3	81.8 ± 1.2	81.8 ± 1.4	82.7 ± 0.9
Immigrant to Canada (% yes)	7.8 ± 1.0	9.7 ± 0.8	18.8 ± 1.3	29.5 ± 1.1 *

* Significant at 0.05 level of significance using chi-squared for categorical variables and t-test for continuous variables. RTEC consumers were compared to RTEC non-consumers separately for children and adults. ^1^ All data are weighted and bootstrapped to obtain estimates at the Canadian population level. Data source: 2015 Canadian Community Health Survey-Nutrition. RTEC consumers were defined as those individuals reporting any quantity of RTEC consumption on day 1 of their 24-h recall. ^2^ Smoking data was only available for children aged ≥12 years, ^3^ For children, the variable reflects whether an adult member of the household is a university graduate. ^4^ Marital status only for age ≥19 years. ^5^ For those age 5–18 years, based on body mass index (BMI) z-score for age and sex.

**Table 2:** Distribution of Ready-To-Eat Cereal Consumers by Regions, Sex, and Age in Canada ^1^.

	**Regions**				
**Characteristics**	**Atlantic (*n*= 545,320)**	**Quebec (*n* = 1,720,854)**	**Ontario (*n* = 2,810,008)**	**Prairies (*n* = 1,339,848)**	**British Columbia (*n* = 938,168)**
Ready-to-eat cereal consumers (% ± SE) *	24.5 ± 1.0	21.9 ± 1.2	21.5 ± 1.1	22.1 ± 1.0	21.2 ± 1.3
	**Ready to Eat Cereal Consumers**				
	**Atlantic**	**Quebec**	**Ontario**	**Prairies**	**British Columbia**
Male RTEC consumers(% ± SE)	24.2 ± 1.5	22.1 ± 1.8	20.1 ± 1.5	22.3 ± 1.4	22.6 ± 1.9
Female RTEC consumers (% ± SE)	24.8 ± 1.3	21.8 ± 1.7	22.8 ±1.4	22.0 ± 1.4	19.9 ± 1.8
	**Ready to Eat Cereal Consumers**				
	**Atlantic**	**Quebec**	**Ontario**	**Prairies**	**British Columbia**
Age 2–18 years RTEC consumers (% ± SE)	25.24 ± 1.36	35.0 ± 2.4	33.9 ± 1.9	36.1 ± 1.8	32.5 ± 2.3
Age ≥19 years RTEC consumers (% ± SE) **	15.23 ± 0.37	19.0 ± 1.4	18.4 ± 1.2	18.4 ± 1.1	18.8 ± 1.5

Data source: 2015 Canadian Community Health Survey-2015. ^1^ All data are weighted and bootstrapped to obtain estimates at the Canadian population level. RTEC consumers were defined as those individuals who reported any quantity of ready-to-eat cereal consumption at day 1 of the 24-h recall. * Significant difference across the Quebec, Atlantic, and Prairies at the 0.05 level of significance using the chi-squared test. ** Significance difference between the two age groups across all regions at the 0.05 level of significance.

**Table 3:** Daily energy and nutrient intake between ready-to-eat cereal (RTEC) consumers and non-consumers by age groups ^1^; data are shown as mean ± SE.

Nutrients	All ages (*n* = 33,633,232)		2–13 Years (*n* = 4,173,477)		13–18 Years (*n* = 2,290,418)		≥19 Years (*n* = 27,169,337)	
	RTEC Consumer	RTEC Nonconsumer	RTEC Consumer	RTEC Nonconsumer	RTEC Consumer	RTEC Nonconsumer	RTEC Consumer	RTEC Nonconsumer
**Energy and Macronutrients**								
Energy (kcal)	1876.2 ± 21.6	1856.7 ± 13.8	1646.3 ± 24.8	1663.5 ± 22.53	2109.0 ± 50.8	2040.7 ± 32.12	1911.0 ± 28.5	1864.0 ± 15.9
Carbohydrates (g)	243.4 ± 2.8	221.4 ± 1.7 *	230.0 ± 3.8	225.9 ± 3.39 *	287.3 ± 7.1	263.2 ± 4.5 *	241.6 ± 3.6	217.9 ± 1.9 *
% Energy from carbohydrates	51.7 ± 0.3	48.0 ± 0.2 *	55.2 ± 0.3	54.1 ± 0.03 *	54.4 ± 0.5	51.4 ± 0.4 *	50.3 ± 0.3	47.1 ± 0.2 *
Total sugars (g)	102.0 ± 1.4	87.3 ± 0.9 *	104.9 ± 2.1	101.9 ± 1.0	127.0 ± 4.0	111.0 ± 2.6 *	97.9 ± 1.8	84.1 ± 0.9 *
Fat (g)	65.5 ± 1.1	69.5 ± 0.7 *	54.7 ± 1.07	58.7 ± 1.0 *	72.1 ± 2.5	76.2 ± 1.5 *	67.7 ± 1.4	70.2 ± 0.8 *
% Energy from fat	30.1 ± 0.2	32.3 ± 0.1 *	29.1 ± 0.3	30.7 ± 0.3 *	29.4 ± 0.5	32.6 ± 0.3 *	30.5 ± 0.3	32.5 ± 0.1 *
Dietary Fibers (g)	19.1 ± 0.2	16.1 ± 0.1 *	15.2 ± 0.3	14.0 ± 0.02 *	18.1 ± 0.5	15.6 ± 0.3 *	20.3 ± 0.3	16.3 ± 0.1 *
Protein (g)	77.5 ± 1.01	77.6 ± 0.6	64.2 ± 1.1	62.8 ± 1.02	83.8 ± 2.7	80.2 ± 1.5	80.4 ± 1.3	78.9 ± 0.7
% Energy from proteins	16.5 ± 0.2	16.8 ± 0.1	15.5 ± 0.2	15.0 ± 0.2	15.9 ± 0.4	15.8 ± 0.2	16.8 ± 0.2	17.0 ± 0.1
Monounsaturated Fatty Acids (g)	23.6 ± 0.4	26.0 ± 0.3 *	19.3 ± 0.4	20.7 ± 0.37 *	25.9 ± 1.01	28.1 ± 0.6 *	24.5 ± 0.6	26.4 ± 0.3 *
Polyunsaturated Fatty Acids (g)	13.4 ± 0.3	14.5 ± 0.1 *	10.1 ± 0.2	11.1 ± 0.3 *	14.0 ± 0.5	15.1 ± 0.3 *	14.2 ± 0.4	14.8 ± 0.2 *
Saturated Fatty Acids (g)	22.6 ± 0.4	22.7 ± 0.2	20.3 ± 0.4	21.4 ± 0.4 *	25.6 ± 1.02	26.0 ± 0.6	22.9 ± 0.5	22.6 ± 0.2
Cholesterol (mg)	217. 4 ± 4.9	270.7 ± 4.03 *	170.0 ± 5.1	205.4 ± 4.04 *	238.0 ± 11.8	265.0 ± 8.0 *	228.1 ± 6.6	278.0 ± 4.7 *
**Vitamins**								
Vitamin B_12_ (mcg)	4.3 ± 0.1	3.8 ± 0.06 *	3.6 ± 0.09	3.3 ± 0.08 *	5.0 ± 0.6	3.9 ± 0.1	4.4 ± 0.1	3.9 ± 0.07 *
Vitamin B_6_ (mg)	1.7 ± 0.03	1.5 ± 0.01 *	1.3 ± 0.03	1.2 ± 0.03 *	1.8 ± 0.07	1.4 ± 0.03 *	1.8 ± 0.03	1.6 ± 0.02 *
Vitamin C (mg)	103.8 ± 2.7	100.1 ± 1.6	109.5 ± 3.9	116.9 ± 3.3	120.8 ± 6.6	111.3 ± 3.6	100.1 ± 3.7	97.4± 1.8
Folate DFE (mcg)	450.1 ± 6.2	434.1 ± 4.05 *	391.8 ± 8.2	391.1 ± 8.5	527.1 ± 20.7	468.8 ± 11.4	456.5 ± 8.11	436.1 ± 4.6 *
Folic Acid (mcg)	129.9 ± 2.4	111.6 ± 1.6 *	123.7 ± 3.7	117.1 ± 3.5 *	176.7 ± 9.6	139.0 ± 4.8 *	125.6 ± 3.01	109.1 ± 1.8 *
Vitamin D (mcg)	5.9 ± 0.1	4.5 ± 0.08 *	6.0 ± 0.1	5.0 ± 0.13 *	6.8 ± 0.2	4.9 ± 0.17 *	5.8 ± 0.2	4.4 ± 0.09 *
Niacin (mg NE)	38.1 ± 0.5	38. ± 0.3	29.6 ± 0.5	28.8 ± 0.5	41.1 ± 1.47	38.4 ± 0.7	40.1 ± 0.6	38.9 ± 0.4
Vitamin A in RAE (mcg)	653.4 ± 15.6	626.8 ± 9.8	595.4 ± 16.5	560.1 ± 14.5	759.8 ± 72.7	614.6 ± 16.3	656.0 ± 20.1	634.7 ± 11.2
Riboflavin (mg)	2.0 ± 0.02	1.8 ± 0.02 *	1.7 ± 0.03	1.6 ± 0.03 *	2.2 ± 0.07	1.8 ± 0.04 *	2.0 ± 0.03	1.8 ± 0.02 *
Thiamin (mg)	1.9 ± 0.03	1.4 ± 0.01 *	1.6 ± 0.03	1.3 ± 0.03 *	2.2 ± 0.06	1.6 ± 0.04 *	2.0 ± 0.04	1.4 ± 0.02 *
**Minerals**								
Sodium (mg)	2613.1 ± 38.6	2716.4 ± 25.3 *	2284.6 ± 48.1	2290.9 ± 38.02	2851.2 ± 74.3	2944.4 ± 57.9 *	2675.0 ± 52.3	2745.2 ± 29.1 *
Potassium (mg)	2769.4 ± 32.7	2585.1 ± 19.03 *	2357.9 ± 38.2	2210.1 ± 35.8 *	2708.1 ± 67.41	2487.0 ± 42.7 *	2893.6 ± 44.09	2632.0 ± 22.1 *
Zinc (mg)	10.6 ± 0.1	10.1 ± 0.1 *	8.5 ± 0.1	8.0 ± 0.14 *	11.2 ± 0.3	10.3 ± 0.2	11.1 ± 0.2	10.3 ± 0.1 *
Calcium (mg)	965.5 ± 14.1	766.6 ± 8.06 *	1018.3 ± 18.8	859.2 ± 16.3 *	1104.5 ± 35.1	921.9 ± 21.1 *	932.7 ± 18.7	745.5 ± 9.08 *
Iron (mg)	14.9 ± 0.1	11.5 ± 0.1 *	12.7 ± 0.2	10.0 ± 0.2 *	16.9 ± 0.5	12.0 ± 0.2 *	15.2 ± 0.2	11.6 ± 0.1 *
Magnesium (mg)	315.9 ± 3.8	292.1 ± 2.4 *	248.1 ± 4.05	229.1 ± 3.5 *	315.2 ± 10.2	275.8 ± 5.1 *	335.2 ± 5.03	300.0 ± 2.8 *

Data source: Canadian Community Health Survey, Nutrition-2015. ^1^ All data are weighted and bootstrapped to obtain estimates at the Canadian population level. RTEC consumers were defined those individuals who reported any quantity of ready-to-eat cereal consumption on day 1 of the 24-h recall. The regression model was adjusted by ethnicity, education, smoking, food security, age, immigration, and energy intake for significant differences between RTEC consumers and non-consumers. NE = niacin equivalents; DFE = dietary folate equivalents; MUFA = monounsaturated fatty acids; PUFA: polyunsaturated fatty acids; RAE = retinol activity equivalents; SFA: saturated fatty acids; Vit: vitamin.. * Intake difference between ready-to-eat consumers and non-consumers for each age group, at a 5% level of significance.

**Table 4:** Represents % contribution of nutrients from ready-to-eat cereal to mean intake for daily nutrients for three age groups among RTEC consumers only ^1^.

Nutrients	2–12 years	13–18 years	≥19 years
	% Contribution from RTEC to Daily Nutrient Intake (*n* = 1,569,205)	% Contribution from RTEC to Daily Nutrient Intake (*n* = 659,855)	% Contribution from RTEC to Daily Nutrient Intake (*n* = 5,125,138)
**Energy and Macronutrients**			
Energy (%)	7.6 ± 0.2	9.4 ± 0.4	9.3 ± 0.2
Carbohydrates (%)	11.4 ± 0.3 L	14.2 ± 0.5	15.3 ± 0.4 * H
Total sugars (%)	8.0 ± 0.3 L	10.5 ± 0.5 H	9.8 ± 0.4 *
Fat (%)	2.7 ± 0.1 L	3.5 ± 0.2	3.6 ± 0.2 * H
Dietary fibers (%)	15.7 ± 0.5 L	19.4 ± 0.8	22.7 ± 0.6 * H
Proteins (%)	4.3 ± 0.2 L	5.4 ± 0.3	5.7 ± 0.2 * H
Monounsaturated Fatty Acids (%)	2.8 ± 0.1 L	3.4 ± 0.3 H	2.9 ± 0.1 *
Polyunsaturated Fatty Acids (%)	4.9 ± 0.2 L	5.8 ± 0.4	6.1 ± 0.2 * H
Saturated Fatty Acids (%)	1.9 ± 0.1 L	2.9 ± 0.3	3.4 ± 0.2 * H
Cholesterol (%)	0.03 ± 0.0 L	0.1 ± 0.0	0.1 ± 0.0 * H
**Vitamins**			
Vitamin B_12_ (%)	0.0 ± 0.0	0.0 ± 0.0	0.01 ± 0.0
Vitamin B_6_ (%)	17.2 ± 0.4	19.7 ± 0.8 H	16.5 ± 0.5 * L
Vitamin C (%)	0.0 ± 0.0 L	0.1 ± 0.1	0.2 ± 0.1 * H
Folate DFE (%)	14.3 ± 0.4 L	15.9 ± 0.8 H	15.3 ± 0.5 *
Vitamin D (%)	6.1 ± 0.4	9.2 ± 1.0 H	5.2 ± 0.4 * L
Folic Acid (%)	29.8 ± 1.1 L	30.3 ± 1.7	33.6 ± 1.1 * H
Niacin (%)	9.4 ± 0.3 L	10.5 ± 0.5 H	10.5 ± 0.3 *
Vitamin A in RAE (%)	<.01 ± 0.0	<0.01± 0.0	<0.01± 0.0
Riboflavin (%)	3.7 ± 0.4 L	8.2 ± 0.8 H	5.6 ± 0.3 *
Thiamin (%)	23.4 ± 0.8 L	29.9 ± 1.2 H	28.8 ± 0.8 *
**Minerals**			
Sodium (%)	7.1 ± 0.2	8.2 ± 0.4 H	6.8 ± 0.2 * L
Potassium (%)	3.5 ± 0.2 L	4.9 ± 0.3	5.7 ± 0.2 * H
Zinc (%)	8.9 ± 0.3 L	10.3 ± 0.6	11.6 ± 0.4 * H
Calcium (%)	5.6 ± 0.2	6.3 ± 0.4 H	5.5 ± 0.2 * L
Iron (%)	32.4 ± 0.7	35.4 ± 1.0 H	32.2 ± 0.6 * L
Magnesium (%)	9.1 ± 0.3 L	10.6 ± 0.6	12.4 ± 0.4 * H

^1^ Data are shown as unadjusted means (SE) and are weighted to the Canadian population. Data source: Canadian Community Health Survey, Nutrition-2015. NE = niacin equivalents; DFE = dietary folate equivalents; RAE = retinol activity equivalents. * Percentage difference of contributions within age groups for contribution to daily nutrients at a 0.05 level of significance. The Hsu multiple comparisons with the best method was applied to identify the lowest (L) and the highest (H) % contributions of nutrient intake [18].

**Table 5:** Represents NRF 9.3 score of RTEC consumers and non-consumers across Canada and the regions ^1^.

Age	Ready-to-Eat Cereal Consumer	Ready-to-Eat Cereal Non-Consumer
	NRF 9.3 Score, SE	NRF 9.3 Score, SE
**National (*n* = 33,633,232)**		
≥2 years (All Canadians)	566.1 ± 3	504 ± 2.1 *
2–12 years	567.5 ± 4.4	513.9 ± 3.7 *
13–18 years	535.5 ± 7.9	468.4 ± 3.9 *
≥19 years	569.6± 4	505.5 ± 2.5 *
**Atlantic (*n* = 2,228,012)**		
≥2 years (All Atlantic residents)	559.4 ± 5.1	478.2 ± 3.89 *
2–18 years	535.8 ± 7.3	471.2 ± 4.9 *
≥19 years	567.5 ± 6.6	479.5 ± 4.5 *
**Quebec (*n* = 7,845,099)**		
≥2 years (All Quebec residents)	561.5 ± 6.7	506.9 ± 3.7 *
2–18 years	558.5 ± 6.4	504.1± 5.3 *
≥19 years	562.8 ± 9.3	507.4 ± 4.3 *
**Ontario (*n* =13,086,112 )**		
≥2 years ( All Ontario residents)	573.6 ± 5.6	508.9 ± 4.1 *
2–18 years	562.1 ± 8.4	496.5 ± 5.3 *
≥19 years	578.7 ± 7.4	511.5 ± 4.9 *
**Prairie (*n* = 6,052,825)**		
≥2 years (All Prairie residents)	552.9 ± 6.4	489.6 ± 4.3 *
2–18 years	551.4 ± 6.2	467 ± 5.2 *
≥19 years	553.6 ± 8.8	494.4 ± 5.3 *
**British Columbia (*n* = 4,421,184)**		
≥2 years (All BC residents)	574.9 ± 8.4	516.3 ± 5.4 *
2–18 years	550.1 ± 10.2	511.9 ± 5.9 *
≥19 years	584.4 ± 11.3	517.1 ± 6.3 *

Data source: 2015 Canadian Community Health Survey-Nutrition. ^1^ All data are weighted and bootstrapped to obtain estimates at the Canadian population level. RTEC consumers were defined as those individuals reporting any quantity of RTEC consumption on day 1 of their 24-h recall. * Represents significant differences of Nutrient-Rich Food Index (NRF 9.3) score between RTEC consumers and non-consumers. The NRF 9.3 score is calculated by the sum of the percentages of daily values of nine nutrients to encourage per 2000 kcal including protein, fiber, vitamin A, vitamin D, vitamin C, calcium, iron, magnesium, and potassium minus the sum of the percentages of maximum recommended values for three nutrients to limit, including saturated fat, total sugar, and sodium [2].

**Table 6:** Percentages of grain consumption from RTEC among RTEC consumers ^1^.

Grain Consumption from RTEC	Age Groups			
	All Canadians (≥2 Years) (*n* = 7354198)	2–12 Years (*n* = 1,569,205)	13–18 Years (*n* = 659,855)	≥19 Years (*n* = 5,125,138)
Contribution of RTEC to total grain consumption (%)	31.0 ± 0.6	25.8 ± 0.8	28 ± 1.0	32.8 ± 0.9 *
Whole grain consumption from RTEC (%)	63.4 ± 1.42	63.7 ± 2.5	60.1 ± 3.6	63.7 ± 1.9

Data source: 2015 Canadian Community Health Survey-Nutrition. ^1^ All data are weighted and bootstrapped to obtain estimates at the Canadian population level. RTEC consumers were defined as those individuals reporting any quantity of RTEC consumption on day 1 of their 24-h recall. * Significant difference between age groups at the 0.05 level of significance.

## Abbreviations

AMPM	Automated multiple-pass method
BMI	Body mass index
BC	British Columbia
CCHS	Canadian Community Health Survey
CAPI	Computer-assisted personal interview
CATI	Computer-assisted telephonic interview
MUFA	Monounsaturated fatty acids
NRF 9.3	Nutrient-Rich Food Index
PUFA	Polyunsaturated fatty acids
RTEC	Ready-to-eat cereal
SFA	Saturated fatty acids
